# Comparative oncology approach to drug repurposing in osteosarcoma

**DOI:** 10.1371/journal.pone.0194224

**Published:** 2018-03-26

**Authors:** Alejandro Parrales, Peter McDonald, Megan Ottomeyer, Anuradha Roy, Frank J. Shoenen, Melinda Broward, Tyce Bruns, Douglas H. Thamm, Scott J. Weir, Kathleen A. Neville, Tomoo Iwakuma, Joy M. Fulbright

**Affiliations:** 1 Department of Cancer Biology, University of Kansas Medical Center, Kansas City, Kansas, United States of America; 2 High Throughput Screening Laboratory, University of Kansas Cancer Center, University of Kansas, Lawrence, Kansas, United States of America; 3 University of Kansas Cancer Center, Kansas City, Kansas, United States of America; 4 Biotechnology Innovation and Optimization Center, University of Kansas, Lawrence, Kansas, United States of America; 5 Institute for Advancing Medical Innovation, University of Kansas Medical Center, Kansas City, Kansas, United States of America; 6 Flint Animal Cancer Center, Colorado State University, Fort Collins, Colorado, United States of America; 7 University of Colorado Comprehensive Cancer Center, Aurora, Colorado, United States of America; 8 Department of Pediatrics, University of Missouri Kansas City, Kansas City, Missouri, United States of America; 9 Department of Pharmacology, Toxicology and Therapeutics, University of Kansas, Kansas City, Kansas, United States of America; 10 Arkansas Children’s Hospital, Little Rock, Arkansas, United States of America; 11 Division of Hematology and Oncology, Children’s Mercy Hospital and Clinics, Kansas City, Missouri, United States of America; University of South Alabama Mitchell Cancer Institute, UNITED STATES

## Abstract

**Background:**

Osteosarcoma is an orphan disease for which little improvement in survival has been made since the late 1980s. New drug discovery for orphan diseases is limited by the cost and time it takes to develop new drugs. Repurposing already approved FDA-drugs can help overcome this limitation. Another limitation of cancer drug discovery is the lack of preclinical models that accurately recapitulate what occurs in humans. For OS using dogs as a model can minimize this limitation as OS in canines develops spontaneously, is locally invasive and metastasizes to the lungs as it does in humans.

**Methods:**

In our present work we used high-throughput screens to identify drugs from a library of 2,286 FDA-approved drugs that demonstrated selective growth inhibition against both human and canine OS cell lines. The identified lead compound was then tested for synergy with 7 other drugs that have demonstrated activity against OS. These results were confirmed with *in vitro* assays and an *in vivo* murine model of OS.

**Results:**

We identified 13 drugs that demonstrated selective growth inhibition against both human and canine OS cell lines. Auranofin was selected for further *in vitro* combination drug screens. Auranofin showed synergistic effects with vorinostat and rapamycin on OS viability and apoptosis induction. Auranofin demonstrated single-agent growth inhibition in both human and canine OS xenografts, and cooperative growth inhibition was observed in combination with rapamycin or vorinostat. There was a significant decrease in Ki67-positive cells and an increase in cleaved caspase-3 levels in tumor tissues treated with a combination of auranofin and vorinostat or rapamycin.

**Conclusions:**

Auranofin, alone or in combination with rapamycin or vorinostat, may be useful new treatment strategies for OS. Future studies may evaluate the efficacy of auranofin in dogs with OS as a prelude to human clinical evaluation.

## Introduction

Osteosarcoma (OS) represents the most common malignant bone tumor with a bimodal peak in adolescents and in those greater than 60 years of age [1]. Overall survival increased significantly between 1973 and 1993, but since that time 5-year survival percentages have remained stagnant at about 60 percent [1]. Attempts have been made to improve survival by the addition of cytotoxic chemotherapy to the traditional backbone of therapy, most recently ifosfamide and etoposide. In patients that had over 10% viable tumor at time of primary tumor resection, additional treatment with ifosfamide and etoposide had no improvement in event free survival [2, 3]. New strategies are needed to expediently move new treatments into the clinical setting that work synergistically with other agents known to effectively treat OS.

OS is classified as an orphan disease as it affects only approximately 800 patients per year in the United States. The cost of new drug discovery for orphan diseases is often prohibitive for pharmaceutical companies [4]. One option to overcome this issue is to repurpose already FDA-approved drugs. We previously demonstrated that non-profit collaborators can advance repurposed FDA-approved drugs to patients much faster and at lower cost than new chemical entities [5]. Furthermore, we showed that drug repurposing opportunities can be rapidly and efficiently achieved in an academic setting [6].

As with drug discovery, the lack of preclinical models that accurately recreate what occurs in humans and serve as effective tools in predicting therapeutic response in cancer patients is a challenge for investigators pursuing drug repurposing [7]. In the case of OS, however, nature provides a model that closely resembles humans. About 10,000 canines a year spontaneously develop OS [8]. There are several similarities between human and canine OS including that it usually involves the limbs, is locally invasive and metastasizes to the lungs [9, 10]. Moreover, metastasis is the major cause of death for both human and canine OS. Indeed, ~80% of canine OS patients demonstrate lung metastasis within six months of limb amputation [11], thus giving translational scientists a great model with rapid read out on drug activity [12]. Finally, comparative studies have demonstrated striking similarities in gene expression between canine and human OS [13].

Here, we employed a comparative oncology approach to drug repurposing in OS. We evaluated an extensive library of FDA-approved drugs for anticancer activity in validated human and canine OS cell lines in parallel. From these studies, auranofin (Ridaura^®^), a FDA-approved oral agent for the treatment of rheumatoid arthritis, was selected for testing its efficacy to suppress OS growth in tissue culture and nude mice, as well as for showing synergistic effects with agents potentially effective in OS therapy. Intriguingly, auranofin had cooperative effects with vorinostat and rapamycin. Our study will advance to a future proof of principle study in canine OS patients and furthermore serve as the rationale for advancing auranofin to human clinical proof of concept studies.

## Material and methods

### OS cell lines

The following cell lines were maintained in Dulbecco’s Modified Eagle’s Medium (DMEM) with 10% fetal bovine serum (FBS) and 1% penicillin-streptomycin: human skin fibroblasts BJ (p53^wt^), human osteoblast hFOB 1.19 (p53^wt^), human OS KHOS/NP (p53^R156P^), Saos2-LM7 (p53^null^), MG-63 (p53^null^), and mouse fibroblasts NIH/3T3 (p53^wt^) were obtained from ATCC, human osteoblasts NHOst (p53^wt^) were obtained from Lonza, canine OS Abrams (p53^R237W,C265F^), and D17 (p53^wt^) were obtained from Douglas H. Thamm (Colorado State University, USA). All cell lines used were authenticated [14–16].

### FDA-approved drug library

The compound management system within the University of Kansas High Throughput Screening (HTS) Laboratory contains 2,286 FDA-approved drugs sourced from Enzo (640 drugs), NIH (446 drugs) and Prestwick (1,200 drugs) compound collections. Compounds are stored at a concentration of 10 mM in dimethyl sulfoxide (DMSO). This library is routinely screened for activity in validated cell-based and biochemical high-throughput screening assays supporting multiple projects, the results of which are contained in an integrated database.

### Primary screen

The cells were plated in 384-well microplates at the following densities: hFOB 1.19 (5,000 cells/well), MG-63 and KHOS/NP (3,000 cells/well), and Abrams (4,000 cells/well), D17 (8,000 cells/well). Each of the 2,286 FDA-approved drugs was transferred to the 384-well assay plates acoustically using Echo 555 (Labcyte) at a final concentration of 2.5 μM. Media and vehicle control wells were included in each assay plate. After 48 hours incubation at 37°C, cytotoxicity was measured on Enspire plate reader (Perkin Elmer) using the luminescence-based CellTiter-Glo reagent (Promega Inc.). All plates included DMSO (0.025%) controls (n = 16). The Z’ scores of five independent screens were >0.7, indicating a good separation of maximum and minimum signals. The Z’ score of >0.5 indicates suitability of the assay for compound screening. Approximately 40 FDA-approved drugs were cytotoxic (defined as >50% inhibition of viable cell proliferation) in some or all of canine and human OS cell lines screened. Thirteen of the FDA-approved drugs that are not used for human or canine OS therapy were selected for further study. These drugs were repurchased as fresh powders and tested for growth inhibition after 48 hours at multiple concentrations across the four human and canine OS cells lines as well as control non-tumor cell lines (hFOB, NIH/3T3, and NHOst) using CellTiter-Glo.as above. The approximate inhibitory concentration at 50% maximal growth inhibition (IC_50_) for each cell line was calculated. For those FDA-approved drugs demonstrating concentration-response, a drug development and regulatory science gap analysis was performed. Drug repurposing opportunities were ranked or prioritized based on: 1) the opportunity to achieve adequate systemic drug exposure following well-tolerated OS patients based on drug-drug interaction potential with current standard-of-care agents; 2) having acceptable safety profile associated with systemic drug administration; and 3) current drug product label supporting rapid translation of the agent to OS patients. Auranofin demonstrated an *in vitro* IC_50_ value ranging from 0.7 to 2.4 μM in all OS cell lines tested. In humans auranofin can achieve steady state mean blood gold levels that approximate 3.5 μM [17].

### Drug combination screens

Doxorubicin, methotrexate, rapamycin, valproic acid, vorinostat, cisplatin, and etoposide were selected for *in vitro* studies applied in combination with auranofin. These seven agents were selected based on their clinical significance as agents are currently used in the upfront treatment of pediatric and canine OS or have demonstrated promising pre-clinical or early phase clinical trial data. *In vitro* IC_50_ values were determined for each of the seven agents individually by determining cytotoxicity over 48 hours of exposure in MG-63 cells, a well characterized, aggressive human OS cell line, using CellTiter-Glo cell viability assays (S1 Fig). The concentration of each agent observed at 50% maximal growth inhibition in 48 hours served as a guideline for defining concentrations of each agent applied in combination with auranofin. Each 384-well plate contained two 11X 14 concentration-matrix blocks, with serial dilutions for auranofin on the X-axis and for these drugs along the Y-axis.

### Drug synergy evaluation

The growth inhibition of each drug alone was first used to identify Highest Single Agent (HSA). GraphPad Prism software (GraphPad Software; Inc, La Jolla, CA) was used to characterize the drug-drug interaction using Bliss independence (combination effect is independent from the action of second agent: Fa+Fb-FaFb), and Chou-Talalay-based Calcusyn software was used to generate concentration-response curves, median-effect plots and a normalized isobolograms. The median effect plots linearize all concentration-effect curves that follow mass action law principle and were calculated from equation: fa/fu = (D/Dm)^m^, where fa is the fraction of cells affected by drug, fu is the fraction of cells unaffected by drug, D is the drug concentration, Dm is the median-effect concentration and m is the slope or kinetic order. The combination index (CI) for each two drug interactions was defined using the following equation: CI = (D)_1_/(Dm)_1_[fa/1-fa]^1/m1^ + (D)_2_/(Dm)_2_[fa/1-fa]^1/m2^, where CI (< 1, = 1 and >1, indicate synergism, additive effect, and antagonism, respectively). The slopes generated by Calcusyn were used to generate normalized isobologram to identify combination data points that fall in the lower left (synergism), on the hypotenuse (additive) or on upper right of the line (antagonism).

### Cell counting assays

This assay was performed to further validate the effects of auranofin on inhibiting proliferation of multiple OS cell lines with minimal effects on that of non-tumor cells. Cells were seeded onto 6-well plates (10,000–30,000 cells per well depending on the cell line, day 0). Twenty-four hours after seeding, cells were treated with 2 different concentrations of DMSO (control) or auranofin. Live cell numbers were counted at days 2, 4, 6, 8, and 10, following trypan-blue staining.

### Western blotting

Western blotting for PCNA, cleaved caspase-3 and vinculin or GAPDH (control) in KHOS/NP and Abrams cell lines was performed in order to examine the effects of auranofin and its combination with vorinostat and rapamycin on their protein expression. KHOS/NP and Abrams cell lines were chosen due to their aggressive behaviors and abilities to cause metastatic disease. Both cell lines contain mutations in the p53 gene [18, 19]. Cells were lysed with radioimmunoprecipitation assay (RIPA) buffer containing phosphatase and protease inhibitors (EMD Chemicals). Cell lysates containing 20–100 μg protein was loaded onto 4–20% tris-glycine gel (Bio-Rad Laboratories, Inc.), separated by electrophoresis, transferred to polyvinylidene fluoride (PVDF) membrane (GE Healthcare Life Sciences), blotted with primary antibodies against specific proteins, and appropriate secondary antibodies conjugated with fluorophores. All blots were analyzed with the LI-COR Odyssey infra-red imaging systems (Lincoln, Nebraska). The following antibodies were used: PCNA (PC-10; Santa Cruz Biotechnology), vinculin (10R-C105a, Fitzgerald), Cleaved-caspase-3 (D3E9; Cell Signaling Technology), GAPDH (FL-335; Santa Cruz Biotechnology), IRDye 680RD goat anti-rabbit IgG, and IRDye 800CW goat anti-mouse IgG (LI-COR).

### Propidium iodide (PI) staining and flow cytometry

In order to examine the effects of auranofin and its combination with vorinostat and rapamycin on cell cycle profiles of OS cells, we performed PI-staining and flow cytometry analysis. Cells were fixed overnight with 70% ethanol at -20°C and stained with PI solution (Life Technologies) in the presence of 62 μg/ml of RNase A, followed by flow cytometric analysis using BD Accuri flow cytometer (BD biosciences, San Jose, CA). Data was analyzed using the FlowJo V10 software (FlowJo LLC, Ashland, Oregon).

### Mice and in vivo tumor growth assay

To perform proof-of-principle studies toward application to canine and human OS clinical investigation, we examined effects of auranofin and its combination with vorinostat or rapamycin on *in vivo* OS growth in nude mice. All mice were purchased from Envigo and were maintained under specific-pathogen-free conditions with regular diet. All experimental procedures were conducted in accordance with the institutional animal welfare guidelines of the University of Kansas Medical Center and we obtained approval from the University of Kansas Medical Center Institutional Animal Care and Use Committee (IAUCUC #2013–2167). Cells (1X10^6^) were subcutaneously injected into 6-week-old female nude-Foxn1^nu^ mice. During injections, mice were gently held and monitored closely for signs of pain or distress. When these signs were noted, the mice were released and the procedure was stopped. When tumors became 3mm in diameter, DMSO, auranofin (0.1 mg/kg), vorinostat (2.5 mg/kg), and rapamycin (0.1 mg/kg), as well as the combination of auranofin with vorinostat or rapamycin were intraperitoneally injected 5 days per week for 3 weeks. Tumor sizes were measured three-dimensionally twice per week during 21 days [20]. Mice were carefully monitored daily with body condition score. When tumors reached approximately 1.5 cm in diameter or mice became moribund, had greater than 20% weight loss or showed <2 for the body conditioned score, they were euthanized utilizing carbon dioxide followed by bilateral thoracotomy.

### Immunohistochemistry (IHC)

After 21 days of treatment with DMSO, auranofin (0.1 mg/kg), vorinostat (2.5 mg/kg), and rapamycin (0.1 mg/kg), as well as the combination of auranofin with vorinostat or rapamycin the tumor tissues were harvested, sectioned at 4 μm, and subjected to IHC by standard procedures. Briefly, sections from the aforementioned tumor tissues were deparaffinized in xylene, rehydrated in grades of alcohol, rinsed in tap water and blocked with 0.3% hydrogen peroxide for 30 min. Antigen retrieval was performed in a steamer with sodium citrate buffer (10 mM sodium citrate, pH 6.0) for 20 min. After blocking in 2.5% normal horse serum for 30 min, sections were incubated with anti-Ki67 (ab15580, 1:2500; Abcam), and anti-cleaved caspase-3 (D3E9, 1:500) for 30 min at room temperature. After washing in PBS, sections were incubated in biotinylated secondary antibody for 30 min. The signal was detected using the Vectastain Elite ABC kit (Vector Laboratories). Pre-immune serum and normal rabbit IgG (Vector Labs, Burlingame) were used as negative controls.

### Statistical analysis

The differences in cell proliferation, percentages of sub-G0/G1 population, and tumor growth between different treatments were analyzed by two-tailed Student’s *t*-tests with GraphPad Prism 5 (GraphPad Software, Jolla, CA). Statistical significance was set at p < 0.05, unless otherwise stated in the text.

## Results

### Primary screening of FDA-approved library identifies auranofin as a potential drug that reduces viability of OS cells

**Fig 1:**
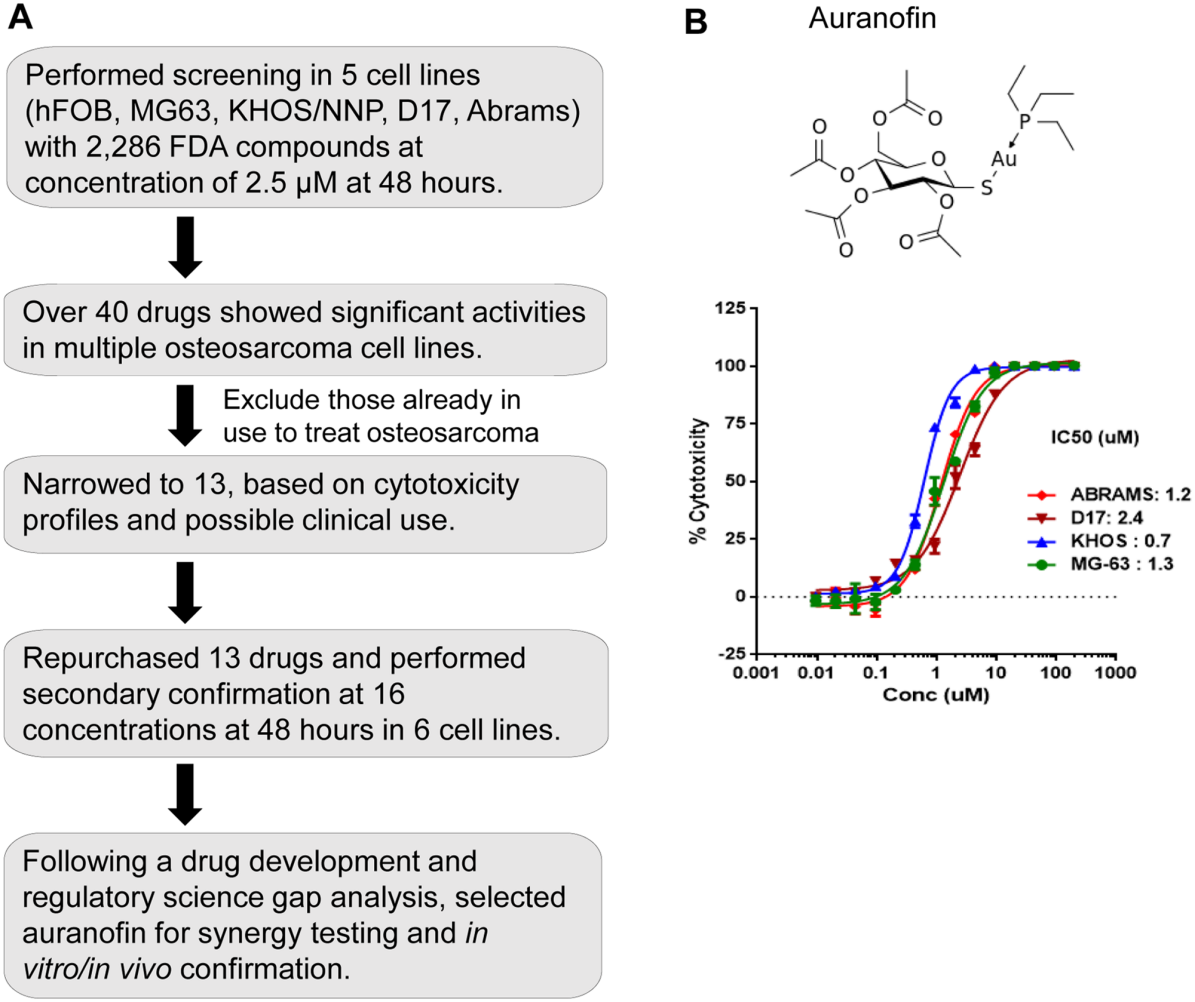
Primary screening of FDA-approved library identifies auranofin as a potential drug for OS therapy. **A.** A diagram of the primary screening to select auranofin. **B.** Auranofin chemical structure (top) and concentration-response curves of auranofin cytotoxic effects on human (MG-63 and KHOS/NP) and canine (Abrams and D17) OS cells (bottom). Graph also includes IC_50_ values for each cell line. Error bars: means ± S.D. from 3-independent experiments.

To identify whether a currently marketed drug contained within our compound library of 2,286 FDA-approved drugs could suppress growth of human and canine OS cells, we performed high-throughput cell viability assays using 2 human (KHOS/NP and MG-63) and 2 canine (D17 and Abrams) OS cell lines, as well as hFOB human osteoblast cell line as a control (S1 Table). Forty drugs showed significant growth suppression as single agents. Thirteen of the 40 drugs identified demonstrated concentration-response relationships resulting in measurable IC_50_ values in human and canine OS cell lines (Fig 1A, Table 1). Auranofin (Ridaura^®^) demonstrated an *in vitro* IC_50_ value ranging from 0.7 to 2.4 μM in all of OS cell lines tested (Fig 1B). We selected auranofin for further *in vitro* combination drug screens based on the following observations: 1) our previous study evaluating auranofin in chronic lymphocytic leukemia patients (NCT01419691) which demonstrates mechanisms of action relevant to the treatment of OS [21], 2) *in vitro* synergistic anticancer activity in combination with other pharmacologic classes of anticancer agents [22], 3) the fact that the current marketed Ridaura^®^ drug product could be readily administered to human and canine OS patients.

**Table 1:** FDA-approved compounds that show inhibition of osteosarcoma cell proliferation.

Compound	Cytotoxicity (% of control)			
	KHOS/NP	MG-63	D17	Abrams
10-Hydroxycamptothecin	82.0	58.0	34.9	66.8
Cerivastatin	72.1	82.3	91.1	74.4
Disulfiram	22.7	8.9	51.8	49.4
Ciclopirox ethanolamine	35.8	20.5	25.4	40.0
Dequalinium dichloride	17.2	35.2	32.2	9.9
Puromycin	95.1	90.2	93.8	70.7
Quinacerine	47.5	28.7	21.7	21.2
Auranofin	70.2	44.4	72.7	86.4
Alexidine dihydrochloride	53.8	38.6	41.0	32.0
Cytarabine	26.4	32.6	15.7	4.4
Antimycin A-HCl	32.8	27.5	48.4	42.1
Amantidine	14.1	23.4	10.6	21.3
Novobiocin a-Na	19.5	26.3	20.7	27.1

**Fig 2:**
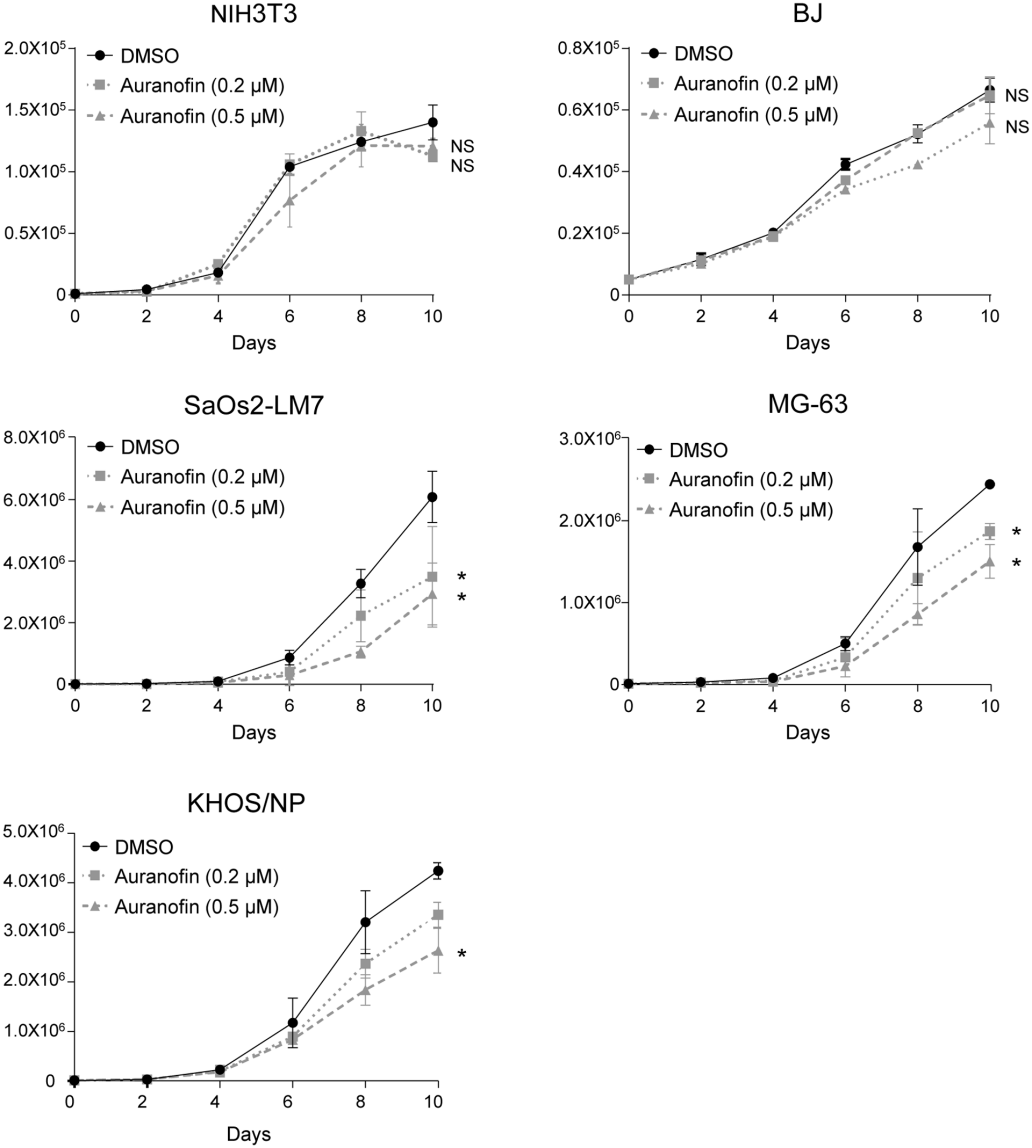
Auranofin preferentially reduces viable cell proliferation of OS cells with minimum effects on non-tumor cells. Mouse and human fibroblast, (NIH/3T3 and BJ, respectively; control; 3x10^4^) as well as human OS cells (Saos2-LM7, MG-63, KHOS/NP, and Abrams; 1x10^4^) were treated with two different concentrations of auranofin (0.2 μM and 0.5 μM) 24 hours after seeding, and the numbers of alive cells were counted every 48 hours for 10 days following trypan blue staining. DMSO was used as a vehicle control. Graphs show numbers of cells on days following treatment with auranofin. Error bars: means ± S.D. from 3 independent experiments. *, P < 0.05; **, P < 0.01; Student’s *t* test. NS: not significant.

We then performed long-term cell proliferation assays by treating 4 aggressive human OS cell lines (MG-63, Saos2-LM7, KHOS/NP, Abrams) and two non-transformed mouse and human fibroblast cell lines (NIH/3T3 and BJ, respectively) with 0.2 μM or 0.5 μM of auranofin (lower than IC_50_ values). Auranofin again showed specificity on OS cell lines to suppress their growth, compared with non-transformed fibroblast cell lines (Fig 2).

### Auranofin shows synergistic effects with vorinostat and rapamycin on viability of MG-63 cells

**Fig 3:**
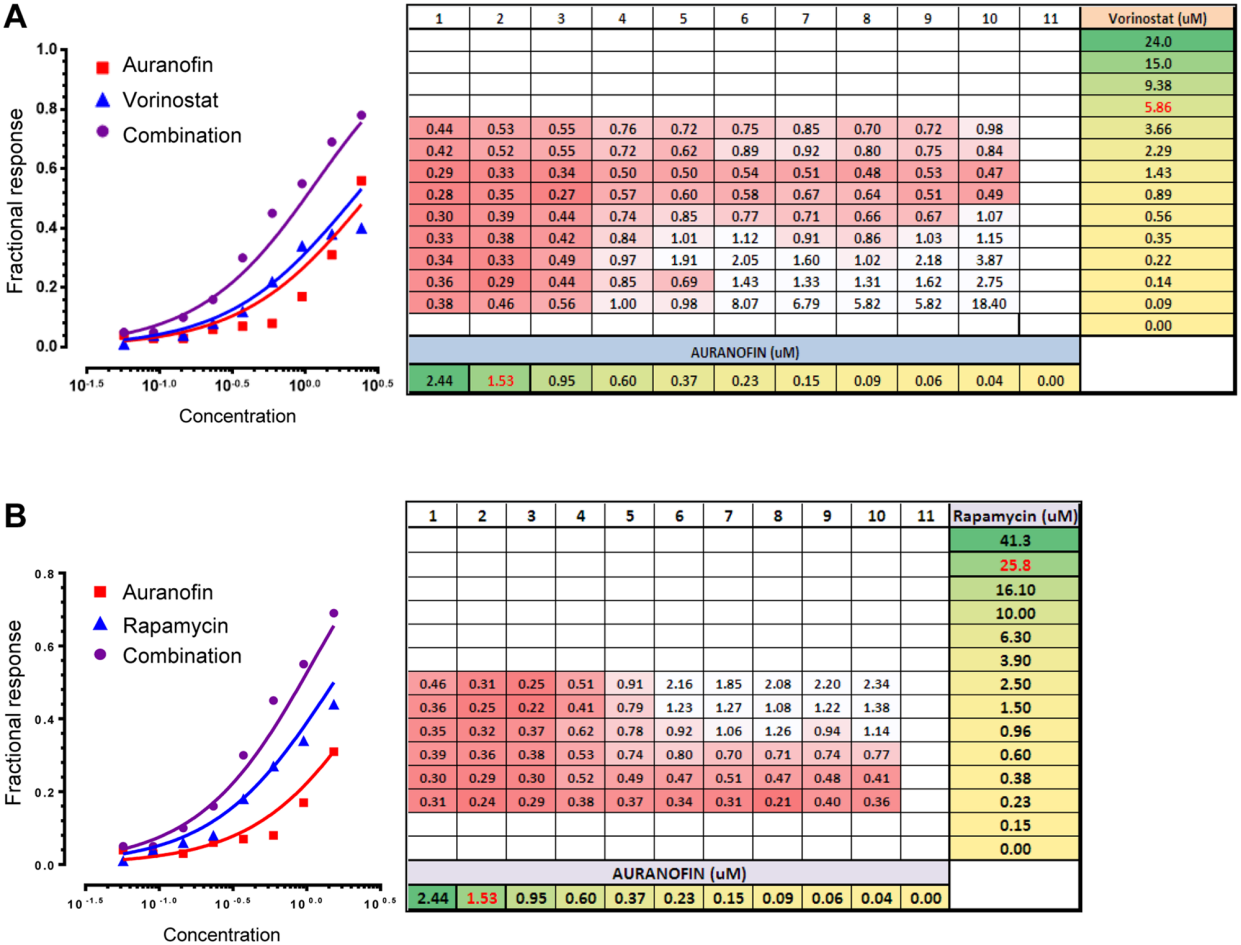
Auranofin shows synergistic effects with vorinostat and rapamycin on the viability of MG-63 cells. MG-63 cells were treated with various concentrations of auranofin along with vorinostat (**A**) or rapamycin (**B**) for 48 hours, and the cytotoxicity was determined. Representative Bliss independence plots are shown on the left. Summaries of all combination indices (numbers) calculated by Chou-Talalay plots using data obtained from varying combination pairs of auranofin and vorinostat or rapamycin (right).

To examine whether auranofin shows cooperative effects with other chemotherapy agents that are currently used for OS treatment or have demonstrated a potential to treat OS in preclinical studies, we treated MG-63 cells with auranofin along with 7 chemotherapy agents, including doxorubicin, methotrexate, rapamycin, valproic acid, vorinostat, cisplatin, and etoposide, at various concentrations to determine combination indices. Of these, vorinostat and rapamycin demonstrated synergistic effects with auranofin, as shown in Fig 3A and 3B, respectively. Thus, hereafter we decided to examine the combinatory effects of auranofin with vorinostat or rapamycin.

### Combination of auranofin with vorinostat or rapamycin synergistically induces apoptosis in OS cells

**Fig 4:**
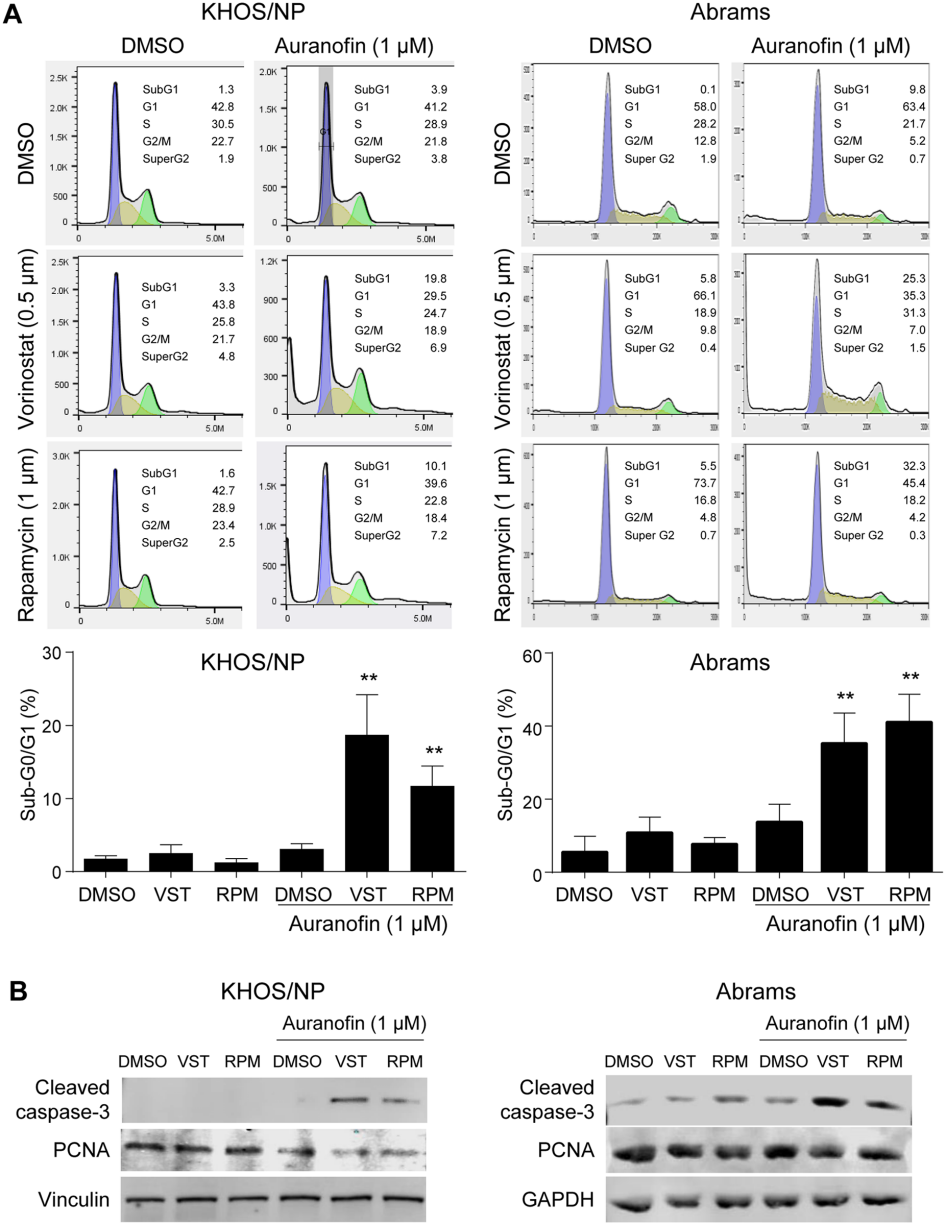
Combination of auranofin with vorinostat or rapamycin synergistically induces apoptosis in KHOS/NP and Abrams cells. **(A)** PI-staining and flow cytometry analysis following treatments of KHOS/NP (left) and Abrams (right) cells with DMSO (control), auranofin, vorinostat (VST), rapamycin (RPM), or indicated combinations for 48 hours. Representative results of cell cycle profiles (top) and a summarized graph showing % of sub-G0/G1 population of the cell cycle following indicated drug treatments (bottom). Error bars: means ± S.D. from three independent experiments. **, P < 0.01; Student’s *t* test. (**B)** Representative images of western blotting for cleaved caspase-3 and PCNA, as well as vinculin or GAPDH as loading controls, using whole protein extracts from KHOS/NP (left) and Abrams (right) cells treated with DMSO, auranofin (1 μM), vorinostat (VST, 0.5 μM), and/or rapamycin (RPM, 1 μM) for 24 hours.

To address the potential mechanism by which auranofin shows cooperative effects on viability of OS cells with vorinostat or rapamycin, we performed propidium-iodide (PI) staining and flow cytometry analyses following treatment of two metastatic OS cell lines KHOS/NP and Abrams with low concentrations (0.5 μM of vorinostat or 1 μM of rapamycin) of these drugs in the absence or presence of 1 μM of auranofin (~50% of IC_50_) for 24 hours [8, 18, 23]. Co-treatment of vorinostat or rapamycin with auranofin significantly increased sub-G0/G1 subpopulation of the cell cycle in KHOS/NP cells, as compared with that with auranofin alone (Fig 4A, left). Similar results were obtained using Abrams cell line (Fig 4A, right). We also confirmed an increase in the expression of cleaved caspase-3 and a decrease in PCNA expression in both KHOS/NP and Abrams cells (Fig 4B). These results suggest that combination of auranofin with vorinostat or rapamycin cooperatively induce apoptosis in KHOS/NP and Abrams cell lines *in vitro*.

### Auranofin, in combination with vorinostat or rapamycin, significantly reduces tumor growth of KHOS/NP and Abrams cells in mice

**Fig 5:**
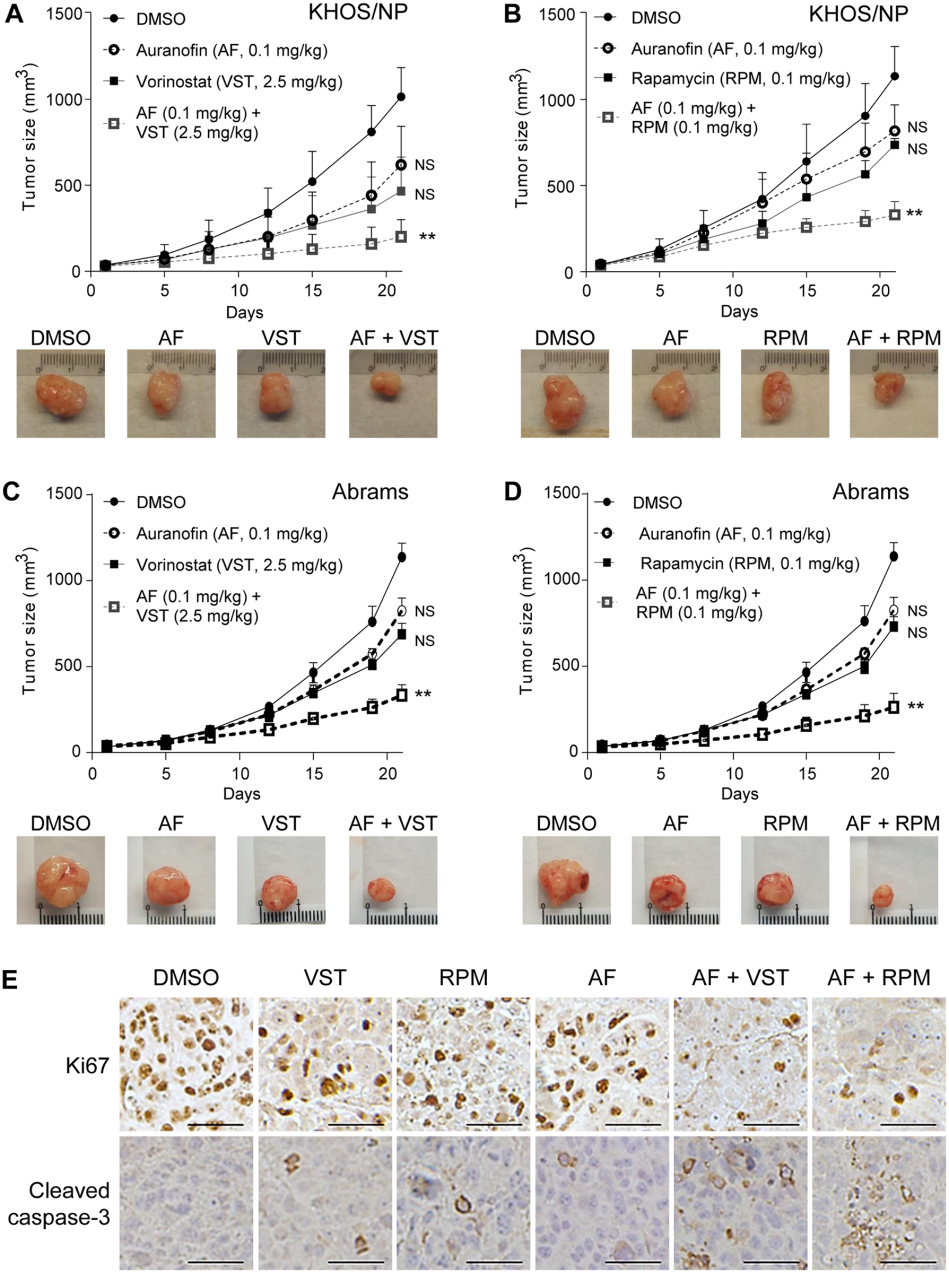
Auranofin, in combination with vorinostat or rapamycin, significantly reduces tumor growth of KHOS/NP and Abrams cells in mice. **A-D.** Tumor formation assays in nude mice subcutaneously injected with KHOS/NP (1X10^6^ (**A**, **B**) or Abrams (1.5X10^6^ (**C**, **D**)) OS cells. When tumors reached 3 mm in diameter, mice were intraperitoneally injected with DMSO or auranofin (AF), along with vorinostat (VST (**A**, **C**), or rapamycin (RPM (**B**, **D**). Tumor sizes were three-dimensionally measured twice a week. Graphs showing sizes of tumors formed in mice (top). Note that the results of DMSO and auranofin alone in (**C)** were also used in (**D)**, since experiments in (**C)** and (**D)** were performed at the same time. Error bars: means ± S.D. (n = 5 animals for each group in (**A** and **B)**; n = 4 animals each group in (**C** and **D**)). **, P < 0.01; Student’s *t* test. NS: Not significant. Representative images of tumors formed in mice at day 21 (bottom). (**E)** Representative images of immunohistochemistry for Ki67 and cleaved caspase-3 using KHOS/NP tumors treated with DMSO or indicated drugs (magnification, 40X). Scale bars, 200 μm.

We next attempted to examine combinatory effects of these drugs on *in vivo* tumor growth. KHOS/NP and Abrams cells were subcutaneously injected into nude mice. When tumors reached 3 mm in diameter, mice were intraperitoneally injected with DMSO or auranofin (AF, 0.1 mg/kg) and/or vorinostat VST, (2.5 mg/kg) or rapamycin (RPM, 0.1 mg/kg) 5 days per week for 3 weeks. Tumor sizes were measured twice per week. Combinations of auranofin with either vorinostat or rapamycin significantly suppressed tumor growth of KHOS/NP cells as compared with single treatments of these drugs (Fig 5A and 5B). Similar results were obtained with Abrams cells (Fig 5C and 5D). During the course of experiments, all three drugs used were well tolerated by tumor-bearing nude mice at doses described above. Moreover, immunohistochemistry analyses using KHOS/NP-tumors revealed that there was significant decrease in Ki67-positive cells and increase in cells positive for cleaved caspase-3 in tumors treated with these drug combinations, as compared with those with DMSO (control) and single-agent treatments (Fig 5E). These results demonstrate cooperative effects of auranofin with vorinostat or rapamycin on the inhibition of *in vivo* OS growth.

## Discussion

In this study, we demonstrate auranofin’s ability to inhibit OS cell growth at an IC_50_ that is achievable in murine models and can be translated into an IC_50_ that is achievable in humans. Auranofin (Ridaura ^®^) is an oral gold complex that is FDA-approved to treat rheumatoid arthritis [24]. It has demonstrated activity in multiple different cancer cell lines including gastrointestinal stromal tumor (GIST) [4], Ewing sarcoma [25], chronic lymphocytic leukemia (CLL) [21], Hodgkin lymphoma [26], and ovarian cancer [27]. Auranofin’s ability to inhibit thioredoxin reductase is one of the main mechanisms by which it causes cancer cell death [25, 28, 29]. Cancer cells generate more reactive oxygen species than normal cells, and therefore they are more dependent upon the systems that regulate the oxidative stress [30, 31]. Thioredoxin reductase is a major cellular anti-oxidant protein [32], and hence by inhibiting this enzyme more oxidative stress occurs in cells. Thus, increased oxidative stress could be a possible mechanism underlying auranofin-induced OS cells death.

One of the major challenges for successfully treating cancer patients is cancer’s resistance to chemotherapy which can be present at time of initial diagnosis or develop over time with treatment [33]. One way to overcome this resistance is to administer drugs that work synergistically [34]. Here, we showed that auranofin has potential synergistic effects with vorinostat and rapamycin. Vorinostat is an inhibitor of histone deacetylases (HDACs), a group of enzymes which are involved in epigenetic modification and interact with multiple transcription factors such as p53 and NF-kB [35]. HDAC inhibitors have demonstrated the ability to decrease OS cell migration *in vitro*, inhibit growth of human OS cells, and induce cell cycle arrest both *in vitro* and in mouse models [36–38]. Rapamycin is an inhibitor of mammalian target of rapamycin (mTOR), a serine/threonine kinase that plays roles in the signaling to a variety of downstream kinases [38]. In murine model of metastatic OS, rapamycin decreases the development of pulmonary metastasis by inhibition of the mTOR/S6K1/4E-BP-1 pathway [39]. Inhibition of this pathway is also found to decrease cell motility [40]. mTOR and its downstream signaling are also found to be effective targets for canine OS cells [41]. Penel-Page *et al*. [42] report that in patients given rapamycin (sirolimus) alone or in combination with other medications, 10 out of the 23 patients have stabilization of disease with 3 maintaining complete remission. Everolimus, also an mTOR inhibitor, in combination with sorafenib, is used to treat patients with relapse or unresectable OS [43]. Even though this study does not meet the target of 6 months progression-free survival (PFS) of 50% or greater, some patients achieve prolonged stabilization of disease/partial response. Currently there are 2 open clinical trials recruiting patients with osteosarcoma to receive metronomic chemotherapy which includes sirolimus; NCT02517918 and NCT02574728. Of note there is currently an open phase I/II trial evaluating the combination of auranofin and sirolimus in adult patients with advanced lung cancer (NCT01737502) providing further evidence that this combination is of clinical interest.

Our *in vivo* studies also showed that there was suppression of OS tumor growth with each drug alone, but tumor growth was significantly inhibited when auranofin was given in combination with either vorinostat or rapamycin. The doses of drugs used in this study were less than those administered in previous studies with other cell lines [21, 39, 44]. Along with suppression of tumor growth, there was also a significant decrease in Ki67-positive cells (consistent with decrease in cell proliferation) and increase in cleaved caspase-3 levels in tumor tissues when treated with combination of auranofin and vorinostat or rapamycin, further supporting the synergism between auranofin and vorinostat or rapamycin. The mechanisms behind their synergism need to be further elucidated.

The major reason we evaluated FDA-approved drugs in both human and canine OS cell lines was to capitalize on canines as a model for OS. OS in canines demonstrates many similarities to human OS [45]. Canines are also treated with local control measures and chemotherapy [46–48]. As in humans, OS in dogs demonstrates high metastatic ability, and the majority of dogs die due to lung metastasis [48]. Currently, evaluation of auranofin in canine cancer patients is on-going in a phase 1 trial to assess the maximum tolerated dose of auranofin in this population. The ultimate goal of our research is to lead to combination therapy with auranofin and vorinostat or rapamycin in human OS patients.

## Supporting information

S1 Table High-throughput cell viability assay results. (XLSX)

S1 Fig Compound screening algorithm and IC 50 results. (TIF)
